# Relating Advanced Electrospun Fiber Architectures to the Temporal Release of Active Agents to Meet the Needs of Next-Generation Intravaginal Delivery Applications

**DOI:** 10.3390/pharmaceutics11040160

**Published:** 2019-04-03

**Authors:** Kevin M. Tyo, Farnaz Minooei, Keegan C. Curry, Sarah M. NeCamp, Danielle L. Graves, Joel R. Fried, Jill M. Steinbach-Rankins

**Affiliations:** 1 Department of Pharmacology and Toxicology, School of Medicine, University of Louisville, Louisville, KY 40202, USA; kevin.tyo@louisville.edu; 2 Center for Predictive Medicine, Louisville, KY 40202, USA; 3 Department of Chemical Engineering, University of Louisville, Louisville, KY 40292, USA; farnaz.minooei@louisville.edu (F.M.); joel.fried@louisville.edu (J.R.F.); 4 Department of Biology, University of Louisville, Louisville, KY 40292, USA; keegan.curry@louisville.edu; 5 Department of Bioengineering, Speed School of Engineering, University of Louisville, Louisville, KY 40292, USA; sarah.necamp@louisville.edu (S.M.N.); danielle.graves@louisville.edu (D.L.G.); 6 Department of Microbiology and Immunology, School of Medicine, University of Louisville, Louisville, KY 40292, USA

**Keywords:** electrospun fibers, fiber architecture, drug delivery, intravaginal delivery, delivery vehicle

## Abstract

Electrospun fibers have emerged as a relatively new delivery platform to improve active agent retention and delivery for intravaginal applications. While uniaxial fibers have been explored in a variety of applications including intravaginal delivery, the consideration of more advanced fiber architectures may offer new options to improve delivery to the female reproductive tract. In this review, we summarize the advancements of electrospun coaxial, multilayered, and nanoparticle-fiber architectures utilized in other applications and discuss how different material combinations within these architectures provide varied durations of release, here categorized as either transient (within 24 h), short-term (24 h to one week), or sustained (beyond one week). We seek to systematically relate material type and fiber architecture to active agent release kinetics. Last, we explore how lessons derived from these architectures may be applied to address the needs of future intravaginal delivery platforms for a given prophylactic or therapeutic application. The overall goal of this review is to provide a summary of different fiber architectures that have been useful for active agent delivery and to provide guidelines for the development of new formulations that exhibit release kinetics relevant to the time frames and the diversity of active agents needed in next-generation multipurpose applications.

## 1. Introduction

Intravaginal delivery is an effective strategy to improve the localization of antiviral, antibacterial, antifungal, chemotherapeutic, and contraceptive agents within the female reproductive tract (FRT) [1,2]. Relative to oral administration routes, intravaginal delivery localizes agents to the FRT, avoiding both the harsh gastrointestinal environment and hepatic first pass effect. This results in an increase in drug bioavailability within target tissue and corresponding functional activity by decreasing off-target effects and systemic exposure [3]. The inherent characteristics of the FRT, including its large surface area and low enzymatic activity, additionally make the FRT a favorable site for localized active agent administration and targeting [4,5]. 

**Figure 1:**
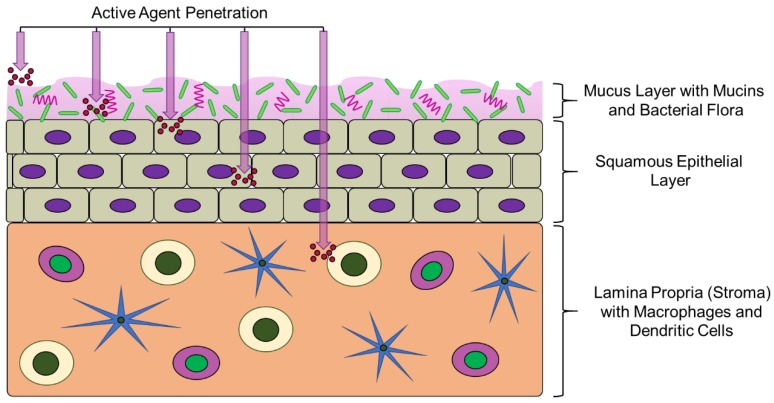
Schematic depicting the structure and specific layers of the vaginal mucosa that can act as a barrier to active agent transport (not to scale). The mucus layer of the female reproductive tract (FRT) frequently sheds and can immobilize active agents (shown in red), leading to decreased efficacy of the administered agents. The bacterial flora normally present within the FRT can also metabolize and degrade agents, further contributing to decreased efficacy. Last, the squamous epithelium can hinder transport to underlying immune cells present near the epithelial surface and/or in the lamina propria.

Although intravaginal delivery offers a variety of advantages to enhance the delivery of active agents [6], challenges unique to the FRT must be overcome to provide efficacious prophylaxis and treatment. One of the most important components of the FRT is the mucus layer, which protects the epithelium and lamina propria from incoming pathogens (Figure 1). However, it can also act as a barrier, impeding therapeutic transport to underlying epithelial and immune cells [7,8]. In addition to these challenges, the frequent shedding and production of cervicovaginal mucus can decrease active agent retention, while bacterial flora, enzymes, and the acidic environment created by beneficial bacteria can contribute to metabolization and degradation of active agents, reducing efficaciousness. 

To address these challenges, intravaginal delivery platforms have been formulated as solid or semi-solid dosage forms that include suppositories, tablets, capsules, gels, rings, and creams to enhance delivery to and retention in the FRT [9,10,11,12,13]. While these dosage forms have enabled high levels of active agent incorporation and localization, these traditionally used delivery platforms still face significant challenges, including difficulty of self-administration, economic feasibility, poor user-compliance, vaginal irritation, the need for frequent administration, and low residence times [14]. Of these platforms, intravaginal rings have provided the “gold standard” for long-term delivery due to their ability to sustain the release of one or multiple active agents for weeks to months, avoid leakage and loss of active agent, and improve drug stability [15,16,17,18,19]. However, some biological agents have difficulty withstanding the high temperature and solvent processes often required for fabrication, limiting their incorporation [20].

More recently, nanoparticles (NPs) have been developed for topical intravaginal delivery due to their ability to encapsulate both hydrophilic and hydrophobic agents and to provide encapsulant stability while enhancing cell specific targeting, transport, and internalization [21,22,23,24,25,26,27,28,29]. However, NPs can experience low intravaginal retention due to mucus shedding, or conversely experience immobilization within the mucus layer, resulting in inadequate transport to underlying tissue [29]. To improve retention and to maximize transport, NPs have been surface-modified [30,31], while carrier solutions with different osmolarities have been explored to increase retention within and penetration of the vaginal lumen [32,33,34]. Despite these efforts, hurdles including low encapsulation efficiency and rapid release of hydrophilic agents have hindered the ability to achieve long-term delivery and retention [35]. Given these issues, other delivery platforms have been investigated that may increase the longevity of active agents within the FRT and improve user adherence while also offering a new dosage form alternative to women. 

Electrospun fibers have recently gained attention for intravaginal delivery due to their ease of use, ability to be fabricated into various geometries and sizes, and tunable release properties [36,37]. They have been considered for sustained-delivery, a characteristic that is often desirable for intravaginal applications, due to their high surface area-to-volume ratio, degree of interconnected porosity, tunable pore sizes, surface-modification potential, interchangeable polymer options, and diverse fiber architectures that enable finer control over the rate, duration, and site of agent release [38]. Electrospun fibers have the additional advantage that they can be fabricated using a variety of natural or synthetic polymers to tailor release properties [39], and these polymer types are typically selected based on their biocompatibility, hydrophobicity, and related degradation properties. 

One of the most significant factors that contributes to active agent release from fibers is the relative hydrophobicity of the selected polymer material [40,41]. In addition to polymer hydrophobicity, the medium (in vitro) or environment (in vivo) surrounding the fiber can impact drug release. Simulated vaginal and seminal fluids, often used to preliminarily assess intravaginal release, may alter the release of agents relative to testing in water or phosphate buffered saline (PBS) (in vitro) or in vivo, due to differences in viscosity, salt, and protein concentrations, as well as pH. Therefore, depending on the degree of polymer hydrophobicity and the environment release it is tested in, the same encapsulated active agent can have distinctly different release profiles, in some cases ranging from hours to months [42,43]. Usually, independent of these conditions, the use of hydrophilic polymers often results in the immediate release of both hydrophilic and hydrophobic active agents due to the high solubility and degradation rate of hydrophilic polymers in aqueous environments [44]. Natural polymers such as collagen, gelatin, chitosan, elastin, and laminin, and synthetic polymers including poly(ethylene oxide) (PEO), polyvinyl alcohol (PVA), and polyvinylpyrrolidone (PVP) are examples of hydrophilic materials that have been fabricated into fibers with micron- and nanometer-scaled properties. In contrast, synthetic hydrophobic polymers including polycaprolactone (PCL), poly(lactic-*co*-glycolic acid) (PLGA), and polyurethane (PU) have demonstrated burst or sustained-release kinetics depending on the hydrophobicity of the incorporated active agent [45,46,47,48,49]. Moreover, synthetic hydrophobic polymers can also serve as a mechanical and structural basis for different fiber architectures in which the release of single or multiple encapsulants may be tailored by using more complex fiber designs or composites. Fiber release rates can also be optimized by adjusting the polymer molecular weight or hydrophilicity, for example, by adding hydrophilic groups such as aliphatic poly(phosphoester) to the polymer structure [50]. Together, these features have enabled the incorporation and release of a variety of antiviral, antimicrobial, and biological agents from fiber scaffolds [51,52,53]. 

Active agent release from polymeric fibers typically occurs via diffusion, polymer degradation, and erosion [41]. When fibers are first administered, solvent or solution diffuses through the porous fiber matrix. Once in contact with the solvent or solution, the polymer matrix swells, loosening polymer chains and enabling the diffusion of active agents, dependent in part on molecular size. Concurrently, the fiber surface may undergo bulk erosion at a rate corresponding to polymer hydrophilicity. These features in combination with the large surface-to-volume ratio of the fibers allows for the increased diffusion of encapsulants relative to diffusion from non-porous bulk materials [54]. Traditionally, fibers have been electrospun as uniaxial fibers or fibers that comprise a single polymer or polymer blend and exhibit homogeneous morphology. Diffusion of active agents from more traditional uniaxial fibers is dependent upon the compatibility of the encapsulant, polymer, and surrounding eluant. In contrast with diffusion, polymer degradation is observed when fibers are exposed to aqueous environments, and polymer bonds are cleaved by either passive hydrolysis or enzymatic reaction [55], resulting in slow degradation of the fiber scaffold. This degradation alters the distance between and size of interconnected pores, thereby impacting the diffusion and release of incorporated active agents. For most synthetic polymers, hydrolysis is the most common mechanism of degradation, although hydrolysis-resistant polymers have been utilized [56], which significantly impact active agent release. As the fibers degrade, they can also undergo surface or bulk erosion, which is dependent upon solvent diffusivity into the fiber, polymer solubility, and overall fiber matrix dimensions [57].

**Figure 2:**
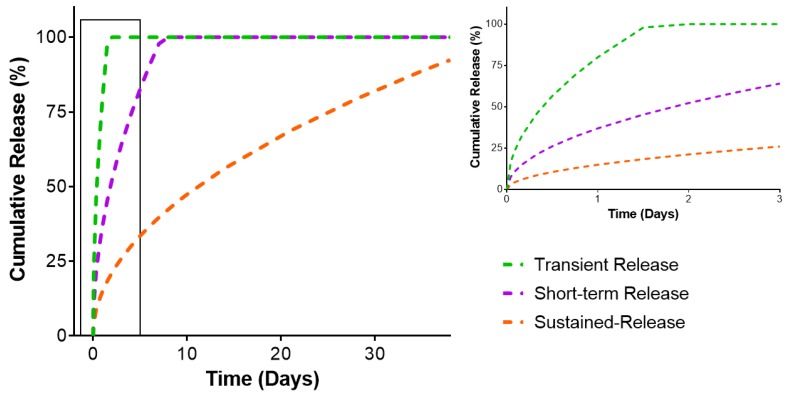
Schematic depicting examples of transient, short-term, and sustained-release profiles.

As a result of these mechanisms and the materials selected, electrospun fibers can tailor the release of encapsulated agents within different durations to achieve immediate (transient or rapid), short-term, or sustained-release. Within this review, we defined release as transient, when the complete release of active agents occurs within 24 h of administration; short-term, when the release occurs from one day to one week; or sustained, when the release of the active agent occurs over a duration of weeks to months. A schematic showing an example of these different potential release profiles is provided in Figure 2. Factors including the electrospinning parameters, polymer materials, fiber architecture, the resulting structure and morphology, and the distribution and amount of incorporated active agent each contribute to the resulting release kinetics and efficacy of delivery [37]. 

Traditional uniaxial electrospun fibers in which each individual fiber is composed of a single cohesive polymer layer were the first fiber architectures to be fabricated [58] and have been utilized in a variety of drug delivery applications over the past decade [36,37,59,60,61]. While uniaxial fibers offer high encapsulation efficiencies, cost-effectiveness, and ease of use, they have suffered from burst release and challenges in tailoring release properties [37,42,62]. These challenges are most evident in achieving the sustained-release of hydrophilic agents, often necessitating hydrophilic polymers to attain high encapsulation efficacy as well as hydrophobic polymers for sustained-release. More complex fiber architectures offer alternative options to address these limitations by combining different polymer types in distinct layers to modulate the release. 

**Figure 3:**
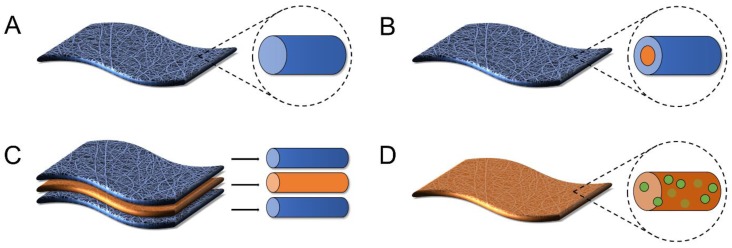
Schematic of different electrospun fiber composites. Diagrams representing (**A**) traditional uniaxial fibers, (**B**) coaxial fibers, (**C**) multilayered fibers, and (**D**) nanoparticle-fiber composites. (**A**) Uniaxial fibers are comprised of a single polymer or polymer blend (shown in blue) that is distributed homogenously throughout the fiber structure. (**B**) In contrast, coaxial fibers contain both core (orange) and shell (blue) layers that are chemically distinct. (**C**) Multilayered fibers result from sequentially electrospinning different fiber layers together or integrating individual layers post-fabrication. (**D**) Finally, nanoparticle-fiber composites consist of hydrophilic or hydrophobic fibers (orange) that encapsulate nanoparticles (green).

While the release characteristics of traditional uniaxial electrospun fibers have been thoroughly reviewed in literature [63,64,65,66,67,68], to our knowledge, there has not yet been a review of the more advanced fiber architectures used to deliver active agents, nor a review that considers the impact these architectures may have on intravaginal delivery applications. Here, we seek to provide an overview of different polymer architectures including coaxial, multilayered, and nanoparticle-fiber composites (Figure 3) as a function of the materials used to construct these architectures that have been utilized in a diversity of health applications. We seek to present different material combinations in these architectures to systematically relate material type and fiber architecture to active agent release kinetics. Last, we explore how lessons derived from these different architectures might be applied in the context of intravaginal delivery to address the needs of future topical sustained-release platforms for a given prophylactic or therapeutic application. The overall goal of this review is to provide a summary of different fiber architectures that have been useful for active agent delivery and to provide guidelines for the development of new formulations based on the knowledge obtained from previous work across other applications. While some of these more complex architectures have only recently been investigated relative to uniaxial fibers, they have demonstrated promise in enabling greater tunability of release and may be useful to apply as new dosage forms for intravaginal delivery and other similar applications.

## 2. Coaxial Electrospun Fibers 

### 2.1. Coaxial Architectures and Properties

**Figure 4:**
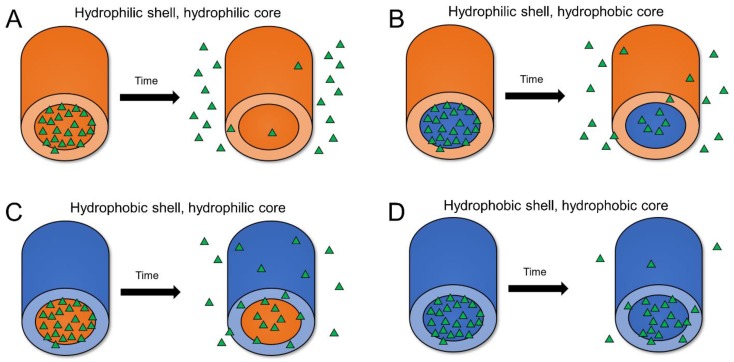
Schematic of anticipated release profiles from different coaxial fiber architectures. Generally, the release of encapsulants from coaxial fibers is dependent on the core and shell hydrophobicity. The release of active agents from coaxial fibers with (**A**) hydrophilic core and shell, (**B**) hydrophobic core and hydrophilic shell, (**C**) hydrophilic core and hydrophobic shell, and (**D**) hydrophobic core and shell are shown. Hydrophilic polymers (shown in orange) typically promote transient release, while more hydrophobic polymers (blue) are typically used to provide short-term or sustained-release.

Coaxial electrospinning, adapted from uniaxial or single axial electrospinning, provides a multicomponent fiber scaffold that easily allows the tunable release of active agents [69,70]. Coaxial fibers are usually comprised of two parts, an outer protective layer or shell and an inner layer or core [71], where encapsulants are typically localized (Figure 3B and Figure 4). Coaxial fibers can provide several advantages relative to uniaxially spun fibers. First, electrospinning the core and shell polymer solutions simultaneously through a coaxial spinneret allows for the design of unique fiber architectures. The thickness and ratios of the core and shell layers can be modulated, providing more reproducible fiber properties with a greater ability to alter encapsulant release relative to other fabrication methods. Additionally, coaxial electrospinning ensures that the active agent in the core phase is protected within harsh physiological environments, such as the female reproductive tract [53]. Furthermore, a variety of materials can be used as either the core or shell to finely regulate encapsulant release (Figure 4) [69,72]. 

Despite these advantages, the added complexity of simultaneously electrospinning two or more polymer phases and the additional interactions between the core and shell solutions requires additional optimization relative to uniaxial electrospinning in terms of selecting compatible polymers and solvents. In addition to the core-shell architecture itself, the release profiles of active agents from coaxial fibers are impacted by solvent choice, polymer-solvent miscibility, the miscibility between core and shell solvents/solutions, solvent volatility, and layer thicknesses [73,74]. Solvent choice has been shown to alter fiber diameter and structure [75], thereby impacting active agent release [76]. Additionally, miscible core and shell solvents/solutions may lead to the partial dissolution of core encapsulants in the shell, whereas, immiscible core and shell solvents may promote material delamination at the core-shell interface, facilitating burst release of the core encapsulant. Therefore, the polymers and solvents for both core and shell layers must be selected based on their individual properties as well as their anticipated interactions [77,78]. In addition, solvent volatility and evaporation rate can affect the distribution and subsequent release of active agents, while the thickness of the polymer shell, polymer composition, and spinning conditions influence encapsulant diffusion rates [79]. Here, we discuss coaxial fibers as a function of their core-shell design, composition, and incorporated active agents to help relate these considerations to the resulting transient, short-term, or sustained-release characteristics.

### 2.2. Release Kinetics from Coaxial Fibers

#### 2.2.1. Transient Release (within 24 h)

##### Hydrophobic Shell—Hydrophilic Core 

Electrospun fibers can be designed to release the active agent immediately or within 24 h of administration if a rapid onset of action is needed for a given application [80]. Moreover, multiple active agents can be incorporated into different layers of a coaxial fiber (core or shell) to provide transient release. 

For application to infectious diseases, coaxially spun fibers that demonstrate burst release followed by lower levels of short-term release may provide on-demand protection against incoming pathogens, increasing the immediate efficaciousness of agents by releasing initially high (burst) concentrations. This type of release can be achieved by employing coaxial fibers comprised of hydrophobic shells and hydrophilic cores. In one study, coaxial and triaxial fiber multi-drug delivery platforms that used PCL as the outermost shell released ~15% and ~80% of two different hydrophilic dyes, keyacid blue and keyacid uranine (KAB and KAU), from the PVP core and PCL shell fibers, respectively, within one hour [69]. In both the coaxial and triaxial fibers, the PVP core containing KAB was protected by the surrounding PCL layer containing KAU, which helped to extend the release of the remaining KAB to 24 h. For the triaxial fibers, a blank PCL layer was electrospun between the outer PCL shell and the inner PEO core. In both the coaxial and triaxial fibers, KAU was released from the shell within 3 h; however, the triaxial fibers better modulated the release of KAB from the core, releasing 50% less during the first hour. The burst release of the KAU dye, observed from both coaxial and triaxial fibers, was attributed to water penetrating the porous fiber shell, allowing transient release. In another example of coaxial fiber design, water-soluble PVP was used as a core with a hydrophobic ethyl cellulose (EC) shell to encapsulate hydrophobic compounds of either quercetin or ketoprofen. Using this architecture, ~75% of both hydrophobic encapsulants were released within 24 h.

##### Hydrophilic Shell—Hydrophobic or Hydrophilic Cores

Similarly, coaxial fibers that have hydrophilic shells can facilitate the rapid release of encapsulated agents with an initial burst release of 1 to 4 h followed by continued transient release within 24 h of administration. One architecture that has been adopted to achieve rapid- or on-demand release from coaxial fibers is a hydrophilic shell in combination with a hydrophilic or hydrophobic core. In one study, zein-PVP core-shell fibers were developed that incorporated the active agent in both the core (zein) and the shell (PVP) layers [81]. Zein, a natural, moderately hydrophobic polymer was used to achieve immediate and transient release of the hydrophobic drug, ketoprofen. A burst release of 43% was observed within the first hour, followed by transient release of the remaining ketoprofen over 10 h. The initial burst release was correlated with rapid dissolution of the hydrophilic shell, while the more transient 10 h release was attributed to the hydrophobic core. In another study, the release profile of a hydrophobic drug, asiaticoside, was compared between coaxial fibers composed of chitosan cores with either a hydrophilic alginate and PVA-blended polymer shell or a hydrophobic centella triterpenes cream shell [82]. The coaxial fiber with the alginate-PVA shell demonstrated 80% more asiaticoside release relative to the centella control within 10 h, which was attributed to the shell hydrophilicity [82]. Additionally, the trend of burst release followed by more gradual transient release was attributed to rapid degradation of the alginate-PVA shell, followed by subsequent degradation of the chitosan core. While this example incorporated a polymer blend (alginate-PVA) as the hydrophilic shell, to be considered a core-shell structure, it should be noted that the material itself needs to be electrospinnable without other polymers. As this example demonstrates, hydrophilic polymers such as PVP, PVA, or PEO can be electrospun alone or in blends to create hydrophilic core and shell layers. 

##### Core-Shell Architectures with Similar Core-Shell Hydrophobicity

Coaxial fibers comprised of both hydrophilic core and shell layers have also been investigated to provide transient release of active agents. For example, coaxial fibers fabricated with a hydrophilic PVP shell and hydrophilic cellulose acetate core were investigated. These coaxial fibers with both a hydrophilic core and shell released 31% of their hydrophobic encapsulant (epicatechin) within 10 min, followed by 80% release after 4 h [83]. 

In addition to the utilization of materials with similar hydrophobicities, coaxial fibers consisting of identical core-shell materials have been fabricated to provide the rapid release of active agents. In one study, fibers with PVP shells and cores were investigated to provide rapid release of the hydrophobic drug, quercetin. The PVP shell-PVP core fibers released quercetin within one minute [84], and this burst release was similarly observed in a separate study that used the same fiber formulation to deliver acyclovir [85]. In another study, the hydrophobic antibiotic, allyltriphenylphosphonium bromide, was incorporated within the core of coaxial fibers, and the volumetric ratios of core-shell solutions were varied to study release. Fibers comprised of zein-zein with core-shell volume ratios greater than 1:2 were found to suppress the burst release of the antibiotic, only releasing 15% within the first hour. In contrast, 35% and 45% of the antibiotic were released from fibers with a 1:1 core:shell volumetric ratio or blended fiber controls over the same duration [86]. In a separate study, a triaxial fiber in which all three layers were comprised of ethyl cellulose provided zero-order release of ketoprofen over 20 h due to the gradual increase in the drug content moving from shell to the core [87]. These studies highlight the role of the active agent distribution within the fiber layers, suggesting that encapsulant localization within the fiber core may enhance release. 

Finally, the release of fluorescently labeled bovine serum albumin (BSA) from core-shell hydrogel nanofilaments composed of a poly(lactide-*co*-ε-caprolactone) (PLCL) shell and *N*,*N*-isopropylacrylamide (NIPAAm)/*N*,*N*′-methylene bisacrylamide crosslinked core was studied. The crosslinker, *N*,*N*′-methylene bisacrylamide, was used to polymerize NIPAAm during the electrospinning process. This study showed that by changing the NIPAAm-crosslinker (*w*/*w*) ratio from 4:1 to 37:1, the release of BSA increased from 0.15 to 0.7 ug/mg over 24 h. However, in the absence of a hydrogel within the core, BSA showed nearly complete release over the same duration. This study demonstrated that the mechanical and corresponding drug release properties could be more finely tailored by altering the NIPAAm-crosslinker (*w*/*w*) ratio [88]. 

##### Stimuli-Responsive Coaxial Architectures

Another method to modulate the release of active agents from coaxial fibers is to integrate stimuli-responsive layers to precisely release agents in response to surrounding physiological conditions [89]. Unlike stimuli-responsive uniaxial fibers, the more complex interactions between the core and shell layers in coaxial fibers can provide increased control of active agent release via pH- or other stimuli-based mechanisms. A variety of natural and synthetic materials have been investigated for their use in pH-responsive applications. In one example, a coaxial fiber comprised of a lecithin-diclofenac sodium core and a Eudragit S100 shell provided the pH-responsive release of ferulic acid for 10 h [90]. Ferulic acid release was facilitated under conditions of neutral pH (pH 7), with minimal release occurring in a more acidic (pH 2) environment. Another pH-sensitive polymethacrylate-based copolymer [90,91,92], Eudragit EPO, was used to fabricate pH-responsive antibacterial fibers. Here, Eudragit EPO cores, which dissolve below pH 5, were used in combination with Eudragit L100 shells, which dissolve at a pH greater than 6. These coaxial fibers provided pH-responsive release for an hour under slightly acidic conditions (pH 6) while demonstrating attenuated release in very acidic conditions (pH 2) [93]. Additionally, two separate studies investigated coaxial fibers comprised of Eudragit S100 shells and PEO cores to stimulate pH-responsive release within the gastrointestinal tract [94,95]. In both studies, the release of hydrophobic indomethacin and hydrophilic mebeverine hydrochloride agents was minimal (~10%) after 2 h under acidic conditions, followed by rapid release for 6 h when switched to neutral conditions (pH 7.4). Coaxial fibers comprised of cellulose acetate phthalate shells with polyurethane cores, as well as gelatin-sodium bicarbonate shells with PLCL cores have also been used to provide similarly rapid pH-responsive release of ciprofloxacin and rhodamine B (Rhd B). These studies demonstrated the potential of coaxial fibers as pH-sensitive delivery systems [96,97].

Coaxial fibers with other stimuli-responsive properties have been investigated for on-demand, rapid release applications. Although studies with other stimuli-responsive systems have been limited, one study investigated the use of self-immolative polymers, or polymers that depolymerize when exposed to specific external stimuli, for rapid stimuli-responsive release [98]. In this study, self-immolative fibers comprised of dibutyltin dilaurate and phenyl (4-(hydroxymethyl)phenyl) carbamate were blended with polyacrylonitrile and used as shells to surround PVP cores. The fibers provided minimal release of KAB dye when incubated in water; however, the fibers depolymerized when exposed to trifluoroacetic acid, resulting in zero-order release of ~40% dye within a week. 

#### 2.2.2. Short-Term Release (One Day to One Week)

##### Hydrophobic Shell—Hydrophilic Core

A key advantage of short-term release specifically for intravaginal delivery is that the burden of frequent or daily administration may decrease, thereby increasing user adherence of prophylactics and therapeutics. Traditionally, hydrophobic materials have been well-suited to provide longer durations of release (depending on the encapsulant) due to their decreased degradation rates in aqueous environments. For more traditional uniaxial hydrophobic fiber platforms, most hydrophobic small molecule drugs or larger macromolecules achieve release for up to one week due to the similar hydrophobic properties of the polymer and encapsulant [6]. This compatibility allows for hydrophobic encapsulants to partition more evenly within and distribute throughout hydrophobic polymers. However, hydrophilic agents, which have low solubility in nonpolar polymers, often partition to the fiber surface, resulting in burst release and suboptimal short-term and/or sustained-release properties. To address this challenge, coaxial fibers in which hydrophilic agents are encapsulated within a hydrophilic core and surrounded by a protective hydrophobic shell can prolong and adjust the release of hydrophilic molecules. 

The use of coaxial fibers with hydrophobic shells and hydrophilic cores has been shown to extend the release of many encapsulants [71,99,100]. In one study, a coaxial fiber comprised of a hydrophobic ethyl cellulose shell with a hydrophilic PVP core was investigated for short-term release. These fibers released maraviroc over a duration of hours to days depending on the thickness of the hydrophobic shell, which was modulated via flow rate and total electrospun volume. The increased thickness of the hydrophobic shell extended encapsulant release from 24 h to five days by increasing the shell-to-core volume ratio from 0.5 to 4 [99]. In another study, a PCL fiber shell surrounding a PVP-graphene oxide blended core was studied. These fibers released 65% of hydrophilic vancomycin hydrochloride within 4 h and attained full release of vancomycin after 96 h [101]. Although this coaxial fiber provided short-term release, the long-term safety of graphene oxide within the FRT is unknown, and further studies are required to assess its safety in intravaginal delivery applications. Finally, a coaxial fiber composed of a synthetic hydrophilic poly-cyclodextrin core and hydrophobic poly(methacrylic acid) shell reduced the burst release of a hydrophilic drug, propranolol hydrochloride, by 50%, and extended release to 180 h relative to the 140 hour release obtained from uniaxial fibers [102]. 

##### Hydrophobic Shell—Hydrophobic Core

In addition to the widely used hydrophobic shell-hydrophilic core coaxial architectures, the use of hydrophobic materials in both the core and the shell layers has also been investigated to provide the short-term release of active agents. In one study, a PCL core surrounded by an outer PCL shell was used to prolong the release of the antibiotic ampicillin. Ampicillin, a hydrophilic compound, normally localizes to the surface of PCL when spun as a uniaxial fiber, resulting in burst release [103]. As an alternative, a 4% (*w*/*v*) PCL solution was used to fabricate an ultra-thin shell to delay release. In addition, the parameters for coaxial electrospinning were modified using dilute sheath solutions to improve the control of fiber diameter and morphology. The resulting coaxial fiber efficiently encapsulated ampicillin and provided short-term release for ~80 h [103]. In another study, coaxial fibers comprised of a zein shell with a PCL core reduced the burst release of the hydrophilic antibiotic, metronidazole, achieving short-term release for more than four days [78]. 

##### Stimuli-Responsive Coaxial Architectures

Coaxial fibers exhibiting stimuli-responsive properties have also been investigated to provide short-term release of active agents. As one example, poly(*N*-isopropylacrylamide), a thermoresponsive polymer, was used as a core layer in combination with an ethyl cellulose and anhydrous ethanol shell solution. At room temperature, poly(*N*-isopropylacrylamide) exhibits hydrophilic properties; however, at temperatures above 32 °C, the polymer demonstrates more hydrophobic characteristics. At room temperature and after 55 h, the fibers released 65% of ketoprofen in PBS, while only 40% of the same drug was released at 37 °C [104]. 

##### Blended Polymers in Coaxial Architectures

Another method of prolonging release is to use blended polymers to formulate coaxial fibers, which can decrease fiber wettability. One study combined gelatin, a natural hydrophilic protein, with the hydrophobic polymer, PCL, to create coaxial fibers with increased hydrophobicity and mechanical stability relative to gelatin alone [105]. In one study, the release of hydrophilic doxycycline was measured from three different fiber architectures—a uniaxial PCL-gelatin blended fiber, coaxial fibers with three different cores (PCL, gelatin, or a PCL-gelatin blend) and a PCL-gelatin blended shell, and a triaxial fiber with both a PCL-gelatin blended core and outer shell and an intermediate gelatin layer. Among these five designs, uniaxial PCL-gelatin blended fibers released the most doxycycline within 24 h (90%), while coaxial fibers with a PCL-gelatin core and shell released the least (50%). Additionally, only coaxial fibers with either a PCL-gelatin or gelatin core prolonged release over five days. Furthermore, the other architectures including the uniaxial PCL-gelatin blend, coaxial fiber with PCL core, and triaxial fibers failed to release doxycycline for more than 30 h. The burst release observed in fibers with PCL cores was attributed to the lack of compatibility between the hydrophobic PCL cores and hydrophilic encapsulant, which caused doxycycline to localize on the core surface. Additionally, the subsequent suboptimal encapsulant release was attributed to low water penetration into the hydrophobic core. These studies demonstrate that utilization of both hydrophobic and hydrophilic polymers alone or as blends can modulate the short-term release of hydrophilic encapsulants due to the variation in the permeability of different layers and core-encapsulant interactions.

#### 2.2.3. Sustained-Release (One Week to Multiple Months)

##### Hydrophobic Shell—Hydrophilic Core

Similar to fibers that provide short-term release, fibers designed for sustained-release commonly use hydrophobic polymers as the outer shell to prevent the fiber from undergoing rapid hydrolysis. Studies have demonstrated that the most promising coaxial architecture to achieve sustained-delivery utilizes a hydrophobic shell and hydrophilic core [6]. A polymer that is frequently used in coaxial fibers to provide sustained-release is poly(lactic-*co*-glycolic acid) (PLGA). In one study, a coaxial fiber composed of a PLGA shell was used to shield a hydrophilic core consisting of tragacanth gum. The encapsulant, tetracycline hydrochloride, served as a model hydrophilic agent. Investigators observed that PLGA (shell)-tragacanth gum (core) coaxial fibers diminished burst release and provided sustained-release of tetracycline hydrochloride for 75 days, releasing 68% of tetracycline hydrochloride during this period [106]. In another study, a PLGA (shell)-polyethylenimine (PEI, core) architecture was used to prolong the release and stability of bone morphogenetic protein-2 plasmid (pBMP2-2). The hydrophilic PEI core was used to encapsulate and retain the bioactivity of pBMP2-2, while the hydrophobic PLGA shell was used as a protective barrier to prolong release. When compared to uniaxial PLGA-PEI blended fibers, the PLGA (shell)-PEI (core) coaxial fiber exhibited both improved bioactivity and prolonged release of the pBMP2-2 plasmid. The coaxial fiber released 80% of the plasmid over 20 days, while the uniaxial fibers released the same amount over seven days [107]. 

Polymers other than PLGA have been used as hydrophobic shells to sustain the release of active agents from coaxial fibers. One study formulated coaxial fibers containing a hydrophilic dextran core and hydrophobic PCL shell. The addition of polyethylene glycol (PEG) to the PCL shell increased the release of the encapsulated BSA by forming pores in the shell layer. Although all fibers released ~20% BSA within the first 24 h, increasing the PEG concentration increased the amount of BSA released over extended durations. Interestingly, all fibers demonstrated sustained-release regardless of PEG concentration; coaxial fibers fabricated with 5% PEG shells released ~60% BSA, while fibers containing 40% PEG shells released 90% BSA over 27 days [108]. In another study, the relationship between PEG (core):PCL (shell) molar ratio and the release of BSA or lysozyme was investigated. The thinnest shell layers with a core:shell molar ratio of 1.59 and a core flow rate of 2 mL/h provided complete release of both encapsulants within 24 days, compared to only 50% release from thicker fibers with a core:shell molar ratio of 0.32 and a core flow rate of 0.6 mL/h. Moreover, the fibers preserved the bioactivity of lysozyme and released BSA over 29 days, with no noticeable differences between BSA and lysozyme release rates [109]. In addition to conventional coaxial spinning, the use of emulsion electrospinning has also been investigated to fabricate coaxial fibers, which can be electrospun using a uniaxial spinneret [70]. One study that used emulsion electrospinning fabricated core-shell fibers composed of a PEG-poly(d,l-lactic acid) shell and methyl cellulose core to minimize the burst release of lysozyme [110]. The release of lysozyme from the core was achieved over 15 days and was dependent on the percent of lysozyme loaded, while the structural integrity and bioactivity of lysozyme was protected by the shell. A later study compared these same coaxial fibers to blended uniaxial fibers composed of PCL and PEG and showed that the coaxial fibers improved sustained-release by releasing ~50% of BSA over 35 days relative to blended fibers, which released ~75% BSA [111]. 

Another study explored the effects of multiple processing parameters, including PEG and PCL concentrations, PEG molecular weight, encapsulant concentration, and fiber diameter, in modulating the release of plasmid DNA (pDNA). Plasmid DNA was encapsulated in a PEI core, and a non-viral gene delivery vector (r-PEI-HA) was incorporated within a PCL shell [112]. An increase in fiber diameter was observed with an increase in all of the three other parameters, while the loading and release of r-PEI-HA were correlated to pDNA concentration in the fiber core and PEG molecular weight. The fibers formulated with high PEG molecular weight and low pDNA concentration exhibited ~30% release of r-PEI-HA over 60 days, while the fibers with high pDNA concentration and low molecular weight PEG completely released pDNA within 60 days.

##### Core-Shell Architectures with the Same Core-Shell Hydrophobicity

Although coaxial architectures with similar core and shell hydrophobicities have been utilized to obtain transient and short-term release, coaxial fibers that use the same materials have been less frequently investigated to provide sustained-release. In one study, PLGA was utilized in both the core and shell layers to investigate the effect on vancomycin and ceftazidime delivery [113]. Both hydrophilic drugs were encapsulated within the core PLGA layer and exhibited similar burst release kinetics within the first day, followed by a second phase of more gradual release over five to ten days. Ninety percent of the antibiotics were released after 11 days, followed by complete release after 25 days, with the more gradual release attributed to the PLGA barrier layer.

### 2.3. Applications for Intravaginal Delivery

The enhanced tunability and versatility provided by the core and shell layers of coaxial fibers make them excellent candidates for intravaginal delivery applications. While uniaxial fibers have been studied for sustained- and stimuli-responsive release of active agents in the FRT [6,114,115,116,117,118,119], they have faced challenges in providing the sustained-release of therapeutically relevant concentrations of individual active agents and effectively modulating the release of multiple agents core [6]. Often, compatibility between the polymer and encapsulant can pose challenges to achieving sustained-release with uniaxial fibers, while coaxial fibers may circumvent this issue by integrating two different polymers, enabling the separation of agents within a compatible polymer formulation (core or shell). Moreover, the additional outer shell can help to modulate release. One can envision that with a coaxial architecture, multiple agents may be delivered against a particular infection to provide a synergistic effect or to provide protection against multiple types of viral or bacterial infections. Together, these features allow for enhanced tunability with the option of providing immediate to short-term release for on-demand applications while also providing long-term release that may be particularly useful in prophylactic or contraceptive applications.

A variety of release kinetics can be attained from coaxial fibers by using different combinations of materials in the core and shell layers. Transient or rapid release of active agents is often accomplished with the use of hydrophilic polymers due to their rapid dissolution in aqueous environments. To achieve short-term release extending to one week, a hydrophilic core in combination with a hydrophobic shell is the most frequently used architecture, enabling the slow dissolution of the shell layer, which acts as a barrier to encapsulant diffusion from the core. For sustained-release applications that require delivery on the order of weeks to months, hydrophobic polymers such as PLGA and PCL are often selected as shell polymers due to their slower degradation kinetics and biocompatibility. Yet, due to the number of parameters involved in the synthesis of coaxial fibers, two similar architectures may still be tailored to perform very differently by altering physical versus chemical properties. An example may be seen in which fibers composed of similar or even the same polymers display very different release rates due to the modulation of shell thickness. In these cases, thinner shells have been shown to provide more transient release, while increasing the shell thickness delays or alters the trend to more gradual release. 

Coaxial fibers have been investigated previously for intravaginal delivery [96,99]. In one study, maraviroc release from coaxial fibers was adjusted by varying the drug loading and solution flow rates to provide release over five days [99]. In addition, pH-responsive coaxial fibers have been fabricated to react in the presence of semen by utilizing the pH-sensitive polymer cellulose acetate phthalate as a shell. The outer shell dissolved immediately after exposure to PBS, promoting pH-responsive release of Rhd B [96].

Although coaxial fibers have shown promise in general drug and initial intravaginal delivery applications, further refinements are required to expand their overall utility. First, compatibility between the solvents of the two polymer electrospinning solutions may limit the potential combinations of core-shell materials and encapsulated agents to achieve successful electrospinning. Additionally, residual solvents from the electrospinning process may interact with and inactivate encapsulated active agents in the core layer. Therefore, while research in coaxial fiber design is still ongoing, other fiber architectures such as multilayered fibers may offer additional advantages to advance intravaginal delivery.

## 3. Multilayered Electrospun Fibers

### 3.1. Multilayered Fiber Architectures and Properties

Multilayered fibers can provide layer-by-layer delivery platforms that are relatively simple and inexpensive to fabricate while allowing for the encapsulation of different active agents within the individual layers. The topology, thickness, and composition of each individual layer can be easily tuned to provide different release properties based on the envisioned application. Moreover, multilayered fibers have been shown to have increased mechanical stability and flexibility compared to coaxial fibers [120]. While the interactions between two or more polymer solution interfaces must be considered for coaxial fibers, multilayered fibers can be fabricated from normally incompatible polymers due to their sequential versus simultaneous fabrication process. 

Electrospun multilayered fibers can be fabricated by sequential layering, stacking, or interweaving fibers [121,122,123]. In sequential layering, the first layer of polymer is electrospun onto a collector, followed by electrospinning additional polymer layers directly onto the same collector. In comparison, “stacking” fibers refers to individually electrospinning each layer separately and subsequently adhering individual layers together post-spin. Stacked fibers share similar physical properties with sequentially-layered fibers, enabling temporally-programmed or spatially-specific delivery of active agents [124]. Finally, the fabrication of interwoven fibers utilizes dual or multiple-syringes to simultaneously electrospin two or more different polymer solutions (usually one hydrophilic and hydrophobic) onto the same collector. In contrast to fibers produced using the sequential layering and stacking processes, which have distinct, separate layers of polymeric fibers, interwoven fibers result from the blending of these different polymer solutions from syringes placed opposite of or adjacent to each other into one integrated layer [125,126,127]. This technique seamlessly integrates both hydrophilic and hydrophobic polymers in a way that prevents unwanted interactions between the electrospun polymer solutions [127,128] while enabling the porosity of the hydrophilic fibers to be altered to more finely tune fiber degradation [129]. Although interwoven fibers do not have a shell layer, the interwoven architecture has been beneficial in promoting cell adhesion and growth and has the potential to more finely modulate active agent release via porosity-based mechanisms for drug delivery applications [130,131].

**Figure 5:**
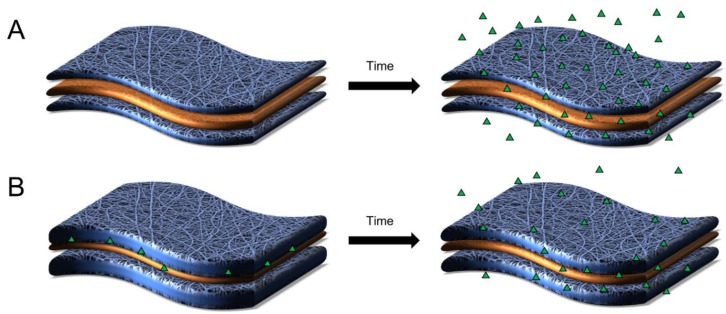
Schematic of anticipated active agent release from multilayered fibers. One method to modulate the release of active agents (shown in green) is to vary the thickness of the outer layer (shown in blue). (**A**) A thin outer layer provides both rapid burst release and limited sustained-release of encapsulants. (**B**) In contrast, increased outer layer thickness can delay the release of some active agents.

Regardless of fabrication technique, multilayered fibers are beneficial in that they can temporally modulate the release of multiple agents from a single delivery platform and can provide additional tunability by modulating the barrier or discrete layers of the multilayered structure (Figure 5). In addition, the ability to impart spatially-specific release—where specific layers of the multilayered fiber possess distinct release profiles—is a key advantage of this architecture. This advantage may be envisioned for intravaginal delivery applications where one layer provides rapid active agent release to the mucus while another layer enables sustained-delivery specific to underlying epithelial or immune cells [121,123]. For interwoven multilayered fibers, studies have shown that the incorporation of a hydrophilic polymer can alter the overall porosity and wettability [129,132,133], while using a hydrophobic outer layer in multilayered fibers (similar to coaxial fibers) can decrease surface wettability and corresponding active agent release [134]. 

While the process of creating multilayered fibers is well established, more work is required to elucidate how each polymer layer impacts release kinetics. Physical properties including the pore size, fiber diameter, and thickness of traditional uniaxial fibers are known to impact the delivery kinetics of active agents from individual layers. Thus, the presence of one or more fiber layers can contribute to the complexity in establishing and predicting the release kinetics of diverse active agents from differently layered architectures. Despite these considerations and complexities, the adoption of different layering techniques to create multilayered fibers can achieve diverse patterns of release for transient, short-term, and sustained-release applications.

### 3.2. Release Kinetics from Multilayered Fibers

#### 3.2.1. Transient and Short-Term Release 

Multilayered fibers have shown promise in providing transient and short-term release of active agents. Conventionally, a hydrophilic layer serves as a reservoir for active agents, while hydrophobic materials provide an outer shell layer to prolong release. One study utilized a multilayered fabrication approach to encapsulate the hydrophobic antibiotic, gentamicin, in a hydrophilic PVA center layer and utilized a PU outer layer to envelop the inner PVA fiber [135]. Three separate fibers were fabricated by altering the thickness of the PU outer layer between 3.4 and 8.1 µm. The release of gentamicin was modulated with the thinnest PU layer (3.4 µm) demonstrating complete release within 1 h, relative to 10% release obtained from the thickest layer (8.1 µm). Furthermore, the thickest PU layer continued to release gentamicin for 24 h. Another study using interwoven electrospun fibers containing PEO and PCL demonstrated that by adjusting the ratio of the two polymers, tunable fiber degradation could be achieved from the resulting changes in pore size and porosity [127]. Although this study investigated interwoven fibers to enhance cell infiltration through the pores, the use of sacrificial fiber layers may be applied to modulate active agent release from the fibers for intravaginal delivery applications [127].

In addition to modulating the outer layer thickness and overall fiber composition, alterations to the number of layers have been shown to impact active agent release. In one study, fibroin-gelatin blended uniaxial fibers exhibited release of trypan blue, fluorescein isothiocyanate (FITC)-inulin, and FITC-BSA within minutes [136]. In contrast, multilayered fibers composed of the same materials extended the release of all three model compounds to 28 days [136]. In another study, dual-release, multilayered electrospun fibers containing the model dyes, 5,10,15,20-tetraphenyl-21*H*,23*H*-porphinetetrasulfonic acid disulfuric acid (TPPS) and chromazurol B, were encapsulated in four-layered PLCL (75:25) fibers. The release rate and duration of the dyes were controlled by the fiber diameter and individual fiber layer thicknesses. Minimal release of both dyes was observed for the first 15 min, followed by a quasi-linear release profile for up to 4 h. However, increasing the thickness of dye-loaded layers resulted in higher quasi-linear release rates due to the reduced density of the fiber surface [137]. In another study, the transient release of ketoprofen was achieved using trilayer fibers composed of two EC outer layers surrounding a center PVP fiber. These fibers provided nearly complete release of ketoprofen within 24 h [121]. Last, asymmetric multilayered polylactide fibers with different designs on each side were fabricated to prevent liver cancer recurrence by promoting one-sided prolonged chemotherapeutic release [138]. The fiber was composed of five poly(lactic acid) (PLA) layers, with each layer serving as either a barrier to release or a drug encapsulating reservoir. In vivo studies in a murine model demonstrated tumor suppression for at least four days, indicating that the multilayered fiber may provide localized chemotherapy for short-term durations [138]. 

Multilayered fibers with stimuli-responsive properties have also been investigated for transient and short-term release applications. In one of the first studies to investigate multilayered architectures, the pH-responsive polymers, poly(acrylic acid) (PAA) and poly(allylamine hydrochloride) (PAH), were electrospun together to create a blended fiber. These fibers were loaded with a low molecular weight cationic molecule, methylene blue, and demonstrated rapid release of methylene blue (~10 min) at a neutral pH (7.4). However, by gradually adjusting the pH from 6 to 2 in aqueous solutions, the step-wise pH-responsive release of methylene blue was achieved over three and a half days. Building upon this work, the effect of coating the fibers with a thermoresponsive polymer blend, poly(*N*-isopropylacrylamide)-PAA, or perfluorosilane was assessed. The addition of the thermoresponsive poly(*N*-isopropylacrylamide)-PAA coating modulated methylene blue release via temperature. Above a critical temperature, the thermoresponsive polymer became insoluble and formed intramolecular hydrogen bonds, which led to the release of methylene blue within 50 min (PBS, pH 7.4). In comparison, coating with perfluorosilane modulated release for up to 20 h at neutral pH. When both the pH-responsive and multiple layers of thermoresponsive polymers were integrated and evaluated at 25 and 40 °C, dye released for a maximum of 10 h regardless of layer thickness [139]. 

#### 3.2.2. Sustained-Release

The ability of multilayered fibers to provide long-term release has been demonstrated in a variety of studies [66,67,140]. In one study, the release of a hydrophobic chemotherapeutic agent, 7-ethyl-10-hydroxycamptothecin (SN-38), was prolonged to 30 days by using a triple-layered fiber in which SN-38 was encapsulated in the center layer and surrounded by two superhydrophobic outer layers consisting of PCL and poly(glycerol monostearate-*co*-ε-caprolactone) [134]. Similar to the trends seen for transient and short-term release from multilayered fibers, increasing the thickness of the outer fiber substantially improved the longevity and amount of drug released. In another study, multilayered fibers comprised of a PCL shell and a PEO/Rhd B core were fabricated to assess the effect of increasing the outer layer thicknesses between 46.1, 68.9, and 186.1 µm [141]. While the thinnest 46 µm layers released 85% of Rhd B in one day, the 68.9 and 186.1 µm layers increased release to 15 and 25 days, respectively. Moreover, the release from the two fibers with the thicker outer layers demonstrated zero-order kinetics, producing gradual, even release of drug with respect to time.

### 3.3. Applications for Intravaginal Delivery

Multilayered fibers have shown promise as a platform to co-deliver or prolong the release of active agents in different environments. The process of creating multilayered fibers is relatively simple, eliminating the more complex set-up and considerations of polymer-solvent interactions between the adjacent, simultaneously spun layers present in coaxial spinning. By removing this complexity of interactions, multilayered fibers can achieve “programmed release” by simply modulating the thickness of each layer.

Multilayered fibers possess other unique features that make them excellent candidates for intravaginal delivery applications. One of the unique strengths of multilayered fibers is that they can provide spatially-specific release in that, unlike other architectures, the individual layers of multilayered fibers can be designed for specific and discrete purposes. For example, one layer may be designed to improve mucoadhesion for enhanced longevity and biocompatibility within the FRT, while another layer may provide release of active agents dependent on its location within the multilayered matrix. Compared to coaxial fibers, the optimization of multilayered fibers is not limited by solvent compatibility, as they can be sequentially spun and assembled post-fabrication. Moreover, multiple individually spun layers can increase the ease of encapsulating multiple types of active agents, which serve mechanistically different roles against a single type of viral infection or as a multipurpose viral-contraceptive or viral-bacterial dosage form. Finally, each fiber layer can be adjusted to have distinct mechanical properties that include tensile strength, porosity, and elasticity, important for comfort and user preference [142].

To date, the use of multilayered fibers for intravaginal delivery has been briefly explored [56,99,123]. In one study, circular sheets of pre-spun PVP and PVP-EC fibers were stacked and annealed via a pressed metal die that was dipped in solvent. The die annealed the edges of the stacked fibers, creating a multilayered fiber with a PVP inner layer surrounded by blended PVP-EC sheaths. Other multilayered fibers were also constructed by folding the outer layers and pressing the seams. Both types of multilayered fibers encapsulated the hydrophilic compound maraviroc and provided biphasic release, exhibiting an initial burst release followed by short-term release for up to five days. Another study from the same group examined tenofovir (TFV) localization within stacked PCL/PLGA fibers. It was found that TFV localization within the multilayered fiber could be predicted by considering the changes in polymer crystalline structure caused by encapsulant-polymer interactions and correlating drug-polymer hydrophilicity [56].

Both multilayered and coaxial fibers have the potential to provide tunable and sustained-release; however, each architecture still faces the challenges surrounding FRT delivery. For example, the interplay between two polymer solutions still needs to be considered for interwoven multilayered (and coaxial) fibers, which may result in challenges to altering active agent release. Additionally, as stated previously, the most significant obstacles to intravaginal delivery are providing a dosage form that can facilitate active agent penetration of mucus and retention and release of therapeutically relevant agent concentrations within the FRT. To improve retention, fibers can be fabricated using polymers or polymer blends that have mucoadhesive properties. However, this longevity is rarely translated to active agents once they have been released from fibers. Thus, new measures may be considered to provide efficacious and sustained-delivery from fibers.

## 4. Composite Nanoparticle-Fiber Delivery Vehicles

### 4.1. Nanoparticle-Fiber Architectures and Properties

Over the past two decades, polymeric NPs have been extensively studied as efficacious drug delivery platforms for a variety of applications. Polymeric nanoparticles are an attractive option for intravaginal delivery relative to traditional delivery platforms such as gels and films due to the tunability of active agent release, ability for surface modification, potential for targeted delivery, enhanced distribution potential, and the often resulting enhanced efficacy of encapsulated agents. Additionally, polymeric NPs have been shown to elicit minimal immune response and to improve the delivery and bioactivity of biologics [29,143,144]. Although metallic nanoparticles have also been explored for use in many drug delivery applications, they have been less commonly administered within the FRT, hence, a more comprehensive review of their applications may be found in [145,146].

Many physicochemical characteristics of NPs can be altered, such as particle size, surface charge, and hydrophobicity, which contribute to their success in achieving sustained-release and localization to target sites [147]. Although NPs have proven to be effective delivery platforms, as discussed in previous reviews [148,149], achieving the prolonged release of active agents can be difficult due to the natural clearance mechanisms of the FRT. In particular, NPs are challenged with retention in the vaginal cavity due to mucus clearance and transport through mucus to underlying tissue [28,150,151]. These challenges may be overcome by incorporating NPs into electrospun fibers, thereby creating a composite delivery vehicle that complements the capabilities of both technologies. One might envision that fibers may act as a reservoir for NPs, improving NP and active agent retention, while the innate fiber porosity can help to more finely tune encapsulant release from NPs relative to the release observed from freely administered NPs or fibers. 

Nanoparticle-fiber composites are dual-component systems that have the ability to alter the release kinetics of active agents from NPs or NPs themselves [152,153]. Often, the active agent of interest is encapsulated within the NPs, which are then preloaded into polymer solutions for subsequent electrospinning. While a variety of inorganic NPs have been incorporated into fibers [154,155,156], concerns still persist regarding the safety of their use relative to polymeric NPs, particularly for intravaginal applications. By utilizing biocompatible polymeric materials for both nanoparticles and fibers, composites may provide safe and prolonged release for clinical applications. 

### 4.2. Release Kinetics from Nanoparticle-Fiber Composites

#### 4.2.1. Transient Release

Nanoparticle-fiber composites have been used to rapidly release NPs and their encapsulated agents. A study was conducted with hydrophilic PVA and PEO fibers that incorporated PLGA NPs that contained the dye, Coumarin 6 [157]. PEO fibers released 90% of NPs within 30 min when immersed in a 50:50 ethanol:PBS solution, followed by additional release (5%) after 3.5 h. In comparison, PVA fibers released approximately 70% of PLGA NPs within 30 min, followed by a decrease in NP release (15%) over 8 h. Slightly slower release over 24 h was observed when PVA fibers were crosslinked prior to NP incorporation. This study highlights that nanoparticle-fiber composites can be used to successfully incorporate NPs and to modulate the transient release of NPs from these composites within aqueous solutions [157]. 

#### 4.2.2. Short-Term Release 

Several studies have utilized nanoparticle-fiber composites to provide the short-term release of active agents. One group explored a composite drug delivery system that encapsulated the antibiotic, erythromycin, in gelatin NPs and free lidocaine hydrochloride within PVA-chitosan blended fibers [158]. Eighty percent of the lidocaine hydrochloride was released from the fibers within 54 h, while 70% of the erythromycin was released after 70 h. In contrast, free gelatin NPs released 90% of erythromycin within the same duration. In a separate study, chitosan-PEO blended fibers containing methoxypolyethylene glycol (mPEG)-b-PLA micelles demonstrated a low initial burst release (15%) of 5-fluorouracil (5-FU), followed by prolonged release (91%) for 109 h [159]. In another study, the release of free hydrophobic naproxen and chitosan nanoparticles containing Rhd B was studied from PCL fiber scaffolds [160]. Rhodamine B exhibited low levels (5%) of burst release, while 30–40% of naproxen was released within the first 2 h. Moreover, after 72 h, only 20% of Rhd B was released, while 60% of naproxen was released. The rapid release of naproxen was achieved via incorporation within the fiber scaffold, while the extended release of Rhd B was obtained and enhanced through nanoparticle-fiber encapsulation. These results demonstrate the utility of nanoparticle-fiber composites in providing the short-term release of multiple agents. 

#### 4.2.3. Sustained-Release

Nanoparticle-fiber composites have also demonstrated long-term release capabilities in several studies. In one study, dual-release nanoparticle-fiber composites were used to mend and treat critically sized calvarial defects in rats [161]. These composites, consisting of PCL-*co*-PEG fibers encapsulating dexamethasone and BSA NPs and loaded with bone morphogenic protein-2 (BMP-2), demonstrated sustained-release of both molecules over 35 days. Another study explored the incorporation of siRNA into chitosan NPs and PLGA fiber composites [153]. In these composites, the release of active siRNA was sustained in vitro, with 95% of siRNA released from the fibers over 32 days, while gene silencing activity was maintained. Sustained-release from nanoparticle-fiber composites was also demonstrated in another study with chitosan-PEO electrospun fibers that were loaded with PLGA NPs encapsulating phenytoin. Nearly complete release of phenytoin from the composite scaffold was achieved over nine days [162]. Lastly, PLA fibers encapsulating chitosan particles provided sustained-release of BSA (45%) for 27 days, while chitosan particles alone released 80% BSA in 14 days [163]. 

In addition to NP incorporation within traditional uniaxial or blended fibers, NPs have been incorporated in more complex fiber architectures to prolong the release of active agents. For instance, the effect of combining a multilayered fiber architecture with nanoparticle-fiber composites was investigated by fabricating alternating layers of poly-l-lactic acid (PLLA) and PCL fibers with layers of PCL fibers encapsulating positively-charged chitosan BSA NPs [164]. The multilayered composite released 80% of the BSA in approximately eight days, whereas the monolayer control released the same concentration of BSA within 24 h.

### 4.3. Applications for Intravaginal Delivery

Composite delivery vehicles containing nanoparticles and fibers have thus far been primarily studied in wound healing and tissue engineering to fabricate scaffolds for tissue regeneration and bone remodeling [86,165,166,167]. However, these platforms may be promising candidates for intravaginal delivery applications due to their structural stability and ability to sustain the release of active agents. In such systems, the fibers may be utilized as a reservoir for NPs to aid in intravaginal retention by helping to decrease NP clearance during shedding. In addition, it is envisioned that, depending on fiber formulation and, importantly, NP size and charge, NP (and active agent) release may be modulated, enabling NPs to traverse mucus and deliver agents to target cells that reside in the epithelium or underlying lamina propria. Similar to other architectures, fiber parameters such as polymer composition and size can be tailored to impact release in combination with altering NP composition, size, and loading within the fiber. 

**Figure 6:**
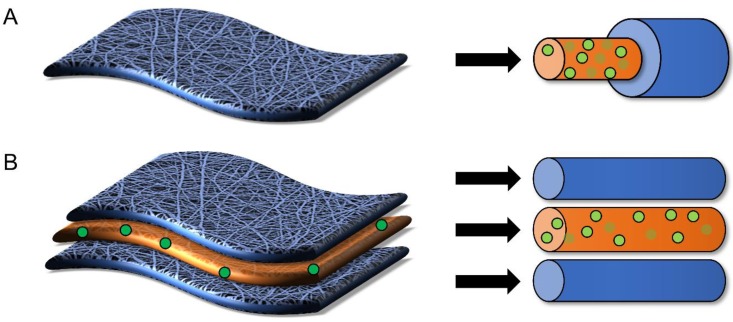
Schematic of electrospun nanoparticle-fiber composites that integrate coaxial and multilayered fiber architectures. (**A**) Coaxial fibers can be fabricated to encapsulate nanoparticles (NPs) within the core fiber, conferring sustained- or delayed-release of active agents that are encapsulated in NPs (shown in green). (**B**) Multilayered fibers that encapsulate NPs can also act as reservoirs for either NP or active agent release.

For intravaginal delivery applications, NPs can impart cell specificity, cell internalization, as well as mucoadhesive or mucopenetrative properties to their encapsulated active agents [14]. Numerous studies have demonstrated the ability of NPs to enhance cell targeting via surface modification [168,169]. Additionally, surface modification can increase cell internalization, which may enhance the transport, subcellular localization, and corresponding efficacy of drugs like tenofovir disoproxil fumarate (TDF), which require cell internalization. Furthermore, the NP surface charge can be modulated to provide either mucoadhesive or mucopenetrative properties that further enhance active agent delivery. Additionally, fibers can be fabricated to encapsulate NPs for sustained-release as well as free agents for rapid release, providing both on-demand and sustained-release in one platform. Finally, nanoparticle-fiber composites, when coupled with coaxial or multilayered fiber architectures, provide an attractive strategy to retain and sustain the release of active agents within the FRT (Figure 6).

As with multilayered fibers, the use of nanoparticle-fiber composites has only recently been investigated for intravaginal delivery. In a proof-of-concept study, rapid-release PEO, PVA, or PVP fibers encapsulated PLGA NPs containing C6 dye or etravirine drug [23]. In this study, composites and free NPs were administered within murine FRTs and assessed for retention and release. The encapsulated nanoparticles exhibited a 30-fold increase in retention in the mouse FRTs relative to free NPs. Furthermore, nanoparticles alone provided transient release of etravirine, while all nanoparticle-fiber composites demonstrated release for up to seven days. To date, this is the only investigation of nanoparticle-fiber composites for use in intravaginal delivery. However, the significant difference in retention and release rate achieved with nanoparticle-fiber composites highlights the immense potential of this architecture for sustained-delivery in the FRT.

Although combining nanoparticles and electrospun fibers into one delivery vehicle has demonstrated potential, challenges exist for this platform. The major concern is related to the concentration of nanoparticles that can be effectively encapsulated within fibers without hindering the ability of the polymer solution to be electrospun [170]. Furthermore, the concentration of active agent may decrease with the use of a coaxial or multilayered architecture, as only specific layers of the fiber will encapsulate NPs. Finally, polymeric NPs are often comprised of the same or similar polymers as electrospun fibers, thus care must be taken to prevent polymer solvents from dissolving the NPs prior to or during the electrospinning process [171]. Moreover, the morphology of NPs may also be adversely affected by electrospinning voltage. These factors limit the combinations of fiber and nanoparticle materials available for composite fabrication. Thus, for composite delivery applications to succeed, polymer choice and electrospinning conditions must be taken into consideration. 

## 5. Future Directions and Discussion

Within the past decade, electrospun fibers have been explored as a multipurpose delivery platform to prevent and treat sexually transmitted infections (STIs). For intravaginal applications, fibers have typically been uniaxially electrospun to release active agents targeted to HIV-1/HSV-2 infections and contraceptive applications. However, other electrospun architectures have been developed that may provide more finely-tuned active agent release, the encapsulation of multiple agents, and longer release durations, desirable for next-generation vehicles. Given this, the goal of this review was to summarize the advancements in electrospun fiber architectures including coaxial, multilayered, and nanoparticle-fiber composites, to meet these needs, and to review their use in other drug delivery applications. We sought to relate different temporal regimens of delivery, including transient (occurring within hours), short-term (spanning hours to one week), and sustained (extending from one week to months), to architectural design and materials selection to help guide the design of future platforms that meet the unique temporal needs of intravaginal delivery.

One of the major challenges facing intravaginal delivery is the lack of user adherence surrounding the administration of current delivery platforms. Several clinical trials have highlighted how a lack of user adherence contributes to decreased efficacy in clinical trials. In both the FACTS-001 and VOICE trials, South African women deemed high risk for HIV-1 exposure were given antiretroviral TFV gels to administer prior to intercourse [93,172]. Despite the known efficaciousness of TFV, the gels provided suboptimal protection against HIV-1 infection, which was attributed to low user adherence of the gels prior to intercourse. Another study examined the efficacy of gels that incorporated the antiviral polysaccharide, carrageenan, in women in Thailand. This study demonstrated similarly disappointing clinical outcomes, with low user adherence considered the most significant reason for the lack of clinical efficacy [173]. Negative outcomes in other trials such as PRO-2000 and cellulose sulfate gel studies, which examined the efficacy of anti-HIV gels in female populations, further validated these studies, highlighting that both user preference and adherence regimens must be considered during product design rather than at the clinical trial stage. As a result of these studies, there has been an increased emphasis to design vehicles that decrease the administration frequency by prolonging active agent release after a single topical application.

In conjunction with improving user adherence, the development of multipurpose delivery vehicles that offer long-term protection against the various stages of a single infection or a diversity of different types of infections is highly desirable [174]. For single infections, a delivery platform may administer multiple agents with different mechanisms of action that target different stages of the viral or bacterial life cycle. However, the increased likelihood of viral co-infections, such as HSV-2 and HIV, as well as bacterial and fungal infections will likely require co-administration of antiviral and antimicrobial agents to be successful. Furthermore, applications that seek to meet both antiviral and contraceptive needs in the same dosage form will require the incorporation of multiple types of agents to expand a platform’s effectiveness. Therefore, a delivery platform that has the capability to release multiple active agents, each over time frames relevant to the application or active agent, will have greater utility and enable more convenient administration schedules based on specific user needs. 

Despite these needs, tailoring the delivery of multiple types of active agents for viral, bacterial, fungal, and contraceptive applications is an ambitious goal given the unique chemical properties of each agent. For example, the antiretroviral TFV and its pro-drug TDF have similar structures and both work as nucleoside reverse transcriptase inhibitors yet possess markedly different hydrophobicities. As such, a delivery platform designed to prolong TFV release may result in different release kinetics of TDF, requiring the formulation of distinct delivery vehicles specific to the selected active agents [56,116,118,175,176]. Furthermore, each active agent may necessitate specific temporal dosing regimens to provide protection or treatment. For example, it may be desirable to administer viral entry inhibitors, which inactivate virions prior to cell entry, over a different time frame than active agents that work inside of cells and need to transport through and localize to target tissue. Several studies have investigated this and have found that more complex and specialized architectures may be useful to achieve temporal delivery goals by tuning the release properties of multiple encapsulants for multiple targets [177,178]. Similarly, for contraceptive applications, on-demand and/or zero-order release with equivalent daily dosing may be desirable for spermicides and hormonal/non-hormonal contraceptives, respectively. Conversely, it may be desirable to deliver active agents such as hormones and small hydrophilic drugs (e.g., etonogestrel and acyclovir) within the same time frame for simultaneous long-term contraception and prevention. However, the drastically different chemical properties of these agents will require more complex solutions to achieve similar release profiles. Given this, multipurpose intravaginal delivery platforms must be tailored to maximize the efficacy of individual active agents, including small molecule drugs, proteins, antibiotics, hormones, and live organisms (e.g., probiotics), to meet the needs of these diverse applications. 

While providing distinct release profiles of different active agents is an important criterion for the development of future intravaginal platforms, to date, intravaginal rings (IVRs) are the only platforms that provide delivery over a duration of weeks to months [179,180,181,182,183]. Furthermore, IVR studies indicate that more complex dosage forms, such as rings with drug-encapsulating pods, may more likely succeed, particularly in challenging delivery scenarios, e.g., achieving the sustained-release of small hydrophilic molecules [177]. These and other studies [177,178,184,185] emphasize the need to offer alternative delivery vehicles for women, with the key lesson that platform architecture must be designed to consider the hydrophobicity and chemical compatibility of the encapsulants in combination with its surrounding materials. 

In addition to the development of fibers with more complex architectures, active agent release and transport from these platforms must be assessed. Tissue mimetics and ex vivo tissues have been used to assess these parameters within the context of intravaginal delivery applications [116,186,187,188,189,190,191]. One of the most common ways in which to assess intravaginal delivery is by using human ectocervical tissue explants derived from patients [187,188,189,190,191]. These explants provide a representative environment in which to measure transport by accounting for the three-dimensional structure of patient tissue. However, patient-specific variations and tissue availability can limit the use of vaginal explants. Given this, organotypic three-dimensional vaginal tissue models such as Epivaginal^TM^ tissue have been created to help evaluate the safety, transport, and efficacy of active agents within an FRT mimetic [192,193]. Other in vitro models have also been developed to explore bacteria and host cell interactions in the reproductive environment [194]. Moreover, within the past decade, new biomarkers and assay endpoints have been identified and studied in different models to more fully assess microbicide interactions with the FRT [195]. The use of tissue models promises to streamline the assessment of future fiber platforms as viable intravaginal delivery platforms.

To date, a variety of studies have developed uniaxial electrospun fibers for intravaginal applications, including HIV prevention [56,115,116,117,118,119,175,176,196,197,198,199]. In these studies, electrospun fibers have demonstrated promising potential for intravaginal applications due to their mucoadhesive characteristics, mechanical properties, and ability to be fabricated in different shapes and sizes [53]. Depending upon the polymer hydrophilicity, traditional uniaxial fibers have been formulated as transient, short-term, or long-term delivery platforms. For the purposes of on-demand and short-term release, many of these studies use hydrophilic fibers, which dissolve or degrade quickly. In contrast, fibers consisting of more hydrophobic materials are expected to persist within the FRT, acting as reservoirs to sustain the release of active agents. We envision (and have observed) that long-term delivery vehicles maintain their structure during the delivery duration of interest and may require physical removal from the FRT, similar to current IVRs. However, one of the key challenges for intravaginal delivery has been to sustain the release of small hydrophilic antiretrovirals due to their rapid diffusion through the porous fiber matrix, solubility in aqueous solutions, and chemical incompatibility with hydrophobic polymer cores [6]. Many of these uniaxial fibers demonstrated burst release of hydrophilic agents followed by short-term release [59,200], partially attributed to the localization of hydrophilic agents on the fiber surface. Compounding this, concerns exist that the subsequent release of active agents may be insufficient to provide complete protection against future infections. While blended uniaxial fibers have been moderately successful in addressing these challenges, more work is required [119].

The primary parameters that impact release from uniaxial fibers are the choice of solvent and polymer. Other factors such as polymer concentration and electrospinning parameters also play a role in attaining different release profiles; however, it is unlikely that these factors alone are sufficient to overcome the challenge of delivering sustained and therapeutically-relevant concentrations of hydrophilic agents. Furthermore, it is difficult to utilize traditional uniaxial fibers for the encapsulation of multiple diverse agents such as large proteins and small drugs. Due to these issues, other electrospinning architectures may be better suited to meet the diverse challenges of intravaginal delivery. 

As discussed previously, coaxial fibers have shown promise for the encapsulation and release of small hydrophilic and hydrophobic molecules, which may be useful for intravaginal delivery applications. The different goals of transient, short-term and long-term release can be achieved by changing the composition and hydrophobicity of core and shell materials as well as by modulating the shell thickness and core:shell ratio. As described, the shell layer can help regulate active agent release, while the core layer is designed to provide optimal compatibility with an encapsulant. For instance, by using pH-responsive polymer shells, an immediate stimuli-responsive release of agents can be achieved when the fiber is in contact with semen. In this scenario, the core layer may be tailored to encapsulate multiple agents, while the shell, comprised of pH-sensitive polymers, retains encapsulants until needed. Another advantage of coaxial fibers is that they can be fabricated to exploit drug-polymer hydrophilicities. For example, a coaxial fiber comprised of a hydrophobic shell and hydrophilic core can be utilized to provide long-term release of hydrophilic compounds. Agent encapsulation into both layers would allow for both transient burst release from the shell due to surface localization and high loading and sustained-release from the core layer. Finally, coaxial fibers can provide release of biological agents such as large proteins. Coaxial cores may be engineered to achieve high protein encapsulation and biocompatibility, while shells can be constructed with porous surfaces, allowing tunable release. This is particularly significant given that many biologics are being investigated as future viral prophylaxes and therapeutics. Although coaxial electrospinning is a more complex process that requires additional optimization, relative to uniaxial spinning, it may enable a versatile platform for transient, short-term, and long-term release [119]. 

Multilayered fibers combine different polymer layers via sequential or post-spinning to incorporate multiple and chemically distinct drugs within specific layers, thereby tailoring the release kinetics for each encapsulated agent. Multilayered interwoven fibers can be utilized to provide transient release using sacrificial layers to encapsulate agents for on-demand applications. The sacrificial layers comprised of hydrophilic polymers would provide on-demand release of agents based on their immediate degradation when exposed to physiological fluids. Active agent release can be further modulated by the number, thickness, and porosity of each fiber layer [201]. Moreover, blank fibers may be incorporated within the multilayers to either act as a physical barrier for sustained-release or for contraceptive purposes. The layer thickness and level of porosity of blank fibers can be conveniently modulated to delay the release of small hydrophilic molecules from the drug-loaded layers, serving to prolong release. Additionally, multilayered fibers have the potential to deliver biologics and non-hormonal contraceptives. These agents, although efficacious, may degrade when exposed to harsh solvents during the electrospinning process. By incorporating these active agents in distinct layers and integrating barrier layers, multilayered fibers can provide long-term release of drugs and biologics while retaining their activities. 

While each of these strategies offers advantages relative to uniaxial spinning, the delivery of active agents may be further enhanced by integrating nanoparticles with fibers. A composite platform may offer a new alternative to address the challenges of intravaginal delivery, such as the maintaining active agent stability, providing cell-specific targeting (via NPs), and enhancing cell internalization. Like electrospun fibers, nanoparticles can be designed to encapsulate virtually any compound. The limitations of nanoparticle-fiber composites mentioned earlier may be overcome by utilizing fibers as a reservoir for both active agents and nanoparticles to release multiple therapeutics. Furthermore, the release rates of encapsulants from both nanoparticles and fibers may be modulated by adjusting the composition of the polymeric scaffold. For on-demand transient release, hydrophilic polymers may be used to enable rapid release of NPs for immediate distribution through and enhanced retention within tissue. In contrast, more hydrophobic fibers may be used to delay the release of NPs or NP-encapsulated agents. Although drug-polymer hydrophobicity is a major contributor to release, other factors such as polymer choice, molecular weight, and crystallinity, as well as solvent choice and electrospinning parameters, also affect the release of agents from fibers.

The application of advanced fiber architectures has only recently been explored in the context of intravaginal delivery. Advanced fiber architectures demonstrate the potential to provide the sustained-release of individual active agents in addition to concurrently providing both transient and sustained-delivery of multiple active agents. These are key advantages over traditional uniaxial fibers, which are challenged with the long-term delivery of small hydrophilic molecules, in addition to providing transient and sustained-release simultaneously. We envision that future fiber architectures will localize active agents within specific sections of the fiber to tailor the release of individual agents independent of other encapsulants. Moreover, we anticipate that future platforms will combine architectures to maximize or complement the advantages of individual platforms. As previous clinical trials have shown, effective protection will be dependent upon fulfilling user preferences, offering convenience, and providing necessary release profiles from one vehicle, which fibers have the potential to realize.
